# Tryptophan and Its Metabolites in Lung Cancer: Basic Functions and Clinical Significance

**DOI:** 10.3389/fonc.2021.707277

**Published:** 2021-08-06

**Authors:** Chenwei Li, Hui Zhao

**Affiliations:** ^1^Department of Respiratory Medicine, The Second Affiliated Hospital of Dalian Medical University, Dalian, China; ^2^Department of Health Examination Center, The Second Affiliated Hospital of Dalian Medical University, Dalian, China

**Keywords:** tryptophan, lung cancer, kynurenine pathway, IDO, TDO

## Abstract

Lung cancer is the most lethal malignancy worldwide. Recently, it has been recognized that metabolic reprogramming is a complex and multifaceted factor, contributing to the process of lung cancer. Tryptophan (Try) is an essential amino acid, and Try and its metabolites can regulate the progression of lung cancer. Here, we review the pleiotropic functions of the Try metabolic pathway, its metabolites, and key enzymes in the pathogenic process of lung cancer, including modulating the tumor environment, promoting immune suppression, and drug resistance. We summarize the recent advance in therapeutic drugs targeting the Try metabolism and kynurenine pathway and their clinical trials.

## Introduction

Lung cancer (LC) is one of the most common malignancies worldwide and has a high mortality rate (1). Previous studies have shown that lung carcinogenesis is attributed to the gain-functional mutation of several cancer-associated genes, including the epidermal growth factor receptor (EGFR), Kirsten rat sarcoma viral oncogene homolog (KRAS), and v-raf murine sarcoma viral oncogene homolog B1 (BRAF) (2–4). Actually, therapeutic drugs targeting these molecules have been demonstrated to prolong the survival of LC patients, particularly for non-small cell lung cancer (NSCLC) patients. However, therapeutic efficacy of these drugs is limited due to rapid development of drug resistance in LC patients (4–6). Therefore, other effective treatments are urgently needed. Currently, cancer has been thought not to be a genetic disease, rather than a metabolic disease, which is associated with tumor immune escape (7, 8). It is well known that tumor cells usually undergo aerobic glycolysis for their glucose metabolism, known as the Warburg effect (9). Moreover, extensive studies have revealed that alternations in metabolisms are not only for glucose, but also for amino acid, lipid, nucleotide, and others in cancer (10). Notably, tryptophan (Try) metabolism is a particularly compelling physiological context in LC because of its complex and multifaceted effect on LC cells and cancer-associated cells in immune escape (11).

Try cannot be synthesized directly by the human body and has the lowest levels in the human body among 20 essential amino acids such that it depends on food protein. Similar to other essential amino acids, Try is essential for biosynthesizing cellular protein and formatting cytoskeleton (12). In the circulation, most Try binds to albumin for transportation and only 10%–20% of it remain free amino acid (13, 14). The free Try is mainly degraded through the kynurenine (KYN) pathway and is metabolized to form serotonin or other metabolites (15). Try plays a significantly physiological role in synthesizing proteins. However, the metabolic formation of serotonin and the KYN pathway-mediated metabolism, together with the lack of its endogenous production, may make Try shortage that can impair the protein synthesis. In the KYN pathway, Try is firstly converted to formyl-kynurenine, which is rapidly degraded to KYN by key enzymes of indoleamine-pyrrole 2,3-dioxygenase (IDO)1, IDO2, and tryptophan 2,3-dioxygenase (TDO), particularly by IDO1 (14, 16). Next, KYN is catalyzed into a series of metabolites, including anthranilic acid (AA), kynurenic acid (KA), xanthurenic acid (XA), 3-hydroxyanthranilic (3-HAA), quinolinic acid (QA), and NAD+ (17). In the lung, Try degradation is mainly catalyzed by IDO1 because IDO1 is constitutively expressed in many organs while TDO is predominantly expressed in the liver (18). Previous studies have shown that most Try metabolites in the KYN pathway are associated with the development of many diseases, including cancer. Actually, the IDO1-related Try metabolites are associated with lung cancer development (19, 20). This review aims to summarize the research advance in how Try and its metabolites contribute to the development and progression of LC.

## The Expression and Biological Functions of Try Metabolites In LC

### Try and Its Metabolites in LC

A previous study has indicated that circulating Try levels decrease in patients with lung, gastric, colorectal, breast, and prostate cancer (21). Recent studies using liquid chromatography mass spectrometry (LC-MS) have found that plasma Try and XA levels decrease and 3-HAA increases in 19 NSCLC patients, relative to 10 non-tumor healthy controls (22, 23). Similarly, high-performance liquid chromatography-fluorescence detection (HPLC-FD) or gas chromatography mass spectrometry (GC-MS) analyses reveal that the concentrations of serum Try in LC patients are significantly lower than that in the controls (24, 25). Moreover, patients with lung adenocarcinoma (LADC) tend to have lower serum Try concentrations than those with lung squamous carcinoma (LSCC), which may be related to its regulatory function in the proliferation and metastasis of different types of cancers (24). However, there is no significant difference in the levels of serum Try during the progression of lung cancer. Accordingly, the levels of serum or plasma Try may be useful for the diagnosis of LC with a specificity of >92% (24). Interestingly, a study reveals that cisplatin-resistant LC cells consume more Try than non-resistant cells (26), suggesting that Try levels may be associated with the development of drug resistance in LC cells. However, how the levels of circulating Try are associated with levels of Try in the tumor microenvironment remains to be investigated.

The decrease in circulating Try may be attributed to several reasons. First, the enhanced expression and activity of Try‐metabolizing enzymes in LC patients can promote Try metabolism, decreasing the levels of Try in the circulation and tumor (27). Second, LC patients may have malnutrition and poor digestion/absorption so that they may intake less Try from foods (24). Last, over-consumption of Try-contained foods may disorder Try metabolism, especially in advanced stage of lung cancer (24, 28) because Try is an essential component for cytoskeleton and protein synthesis in LC.

Decreased circulating Try is a crucial metabolic feature in LC patients. Accordingly, Try levels may combine with other metabolic molecules for diagnosis of LC. Actually, the levels of serum Try, alanine, valine, isoleucine, histidine, and ornithine have a diagnostic value for NSCLC with an area under the receiver-operator characteristic (ROC) of >0.80, and effectively discriminate neoplastic patients from healthy subjects (29, 30). LC patients display decreased levels of serum Try, threonine, citrulline, and histidine and increased proline, isoleucine, phenylalanine, and ornithine, leading to an area under curve (AUC) of 0.80, but the Try metabolite profile does not distinguish different pathological types of LC (29, 31). Consistently, HPLC-FD analysis indicates that a combination of six metabolites [L-tryptophan, hypoxanthine, inosine, indoleacrylic acid, acylcarnitine C10:1, and lysoPC (18:2)] effectively separates NSCLC patients from non-tumor subjects with an AUC of 0.99 (32).

### IDO1, IDO2, and TDO in LC

#### IDO1

The IDO1 is a key enzyme in the KYN pathway, particularly in the lung. Previous studies have detected IDO expression in tumor cells, blood vessels, stromal cells of NSCLC patients, as well as in dendritic cells (DCs) in the tumor environment and tumor-related lymph nodes in patients with LC (33). However, the function of IDO1 in endothelial cells has yet been understood (34). The expression of IDO promotes KYN accumulation, which may dilate blood vessels (35). Accordingly, it is possible that IDO1 deficiency may reduce vascular-related adverse reaction of some therapeutic drugs pharmacologically (35). Besides, IDO1 mRNA transcripts are upregulated in lung tissues (36) and the serum KYN : Try ratio (KTR), an indicative of IDO activity, is greater in LC patients than healthy subjects (37), supporting the notion that higher KTR is associated with increased risk for LC (38), especially for LSCC in heavy smokers, because AhR (aryl hydrocarbon receptor) activates the carcinogenesis pathway of benzo(a)pyrene (BaP), a strong lung carcinogen derived from tobacco smoking (39). High levels of IDO1 expression can enhance LC cell invasion *in vitro* and distant metastasis into the brain, liver, and bone *in vivo*, while IDO1 inhibition attenuates their invasion and distant metastasis in rodents (40). Similarly, IDO1 inhibition also inhibits the lung metastasis of breast cancer and improves the survival of tumor-bearing animals (41, 42). Furthermore, IDO1‐deficient mice are partially resistant to cancer growth in a Lewis rat model of lung carcinoma (43).

The activity and expression of IDO1 are associated with diagnosis, prognosis, and therapeutic responses in LC (44–48). IDO1 activity may be a valuable biomarker for evaluating the response to immunotherapy, and its levels may help in choosing therapy for LC patients, who are sensitive to immunotherapy (49). Similarly, increased IDO1 activity is detected in LC patients, who initially respond to immune checkpoint inhibitors (ICIs) and later exhibit cancer progression, leading to a worse prognosis (44). Furthermore, increased IDO1 activity is closely associated with worse survival of NSCLC patients receiving explicit radiotherapy (48, 50). However, these studies were performed in small groups of patients. Therefore, further prospective studies with a larger population are necessary to validate the prognostic value of IDO1 activity in LC patients following radiotherapy. Interestingly, elevated IDO1 expression is associated with better outcome in lung adenosquamous carcinoma patients, especially for those after surgical resection of the tumor (51). The discrepancy may stem from different studying populations. While previous studies mainly focus on patients with unresectable LC and patients receiving chemotherapy or chemoradiation, this study centers on LC patients after radical surgery (51). It is possible that IDO1 activity may have different values in prognosis of different stages of LC following varying therapeutic strategies.

#### IDO2 and TDO

IDO2 and TDO are other key enzymes for Try degradation (52–55). Although the IDO1 and IDO2 genes are highly homologous at human chromosome 8 and tightly connected (56, 57), the IDO2 catalytic activity is much weaker than that of IDO1 *in vitro* and *in vivo* (58). Actually, there is no significant difference in the concentrations of plasma Try and KYN between wild-type and IDO2-deficient mice (59). Human TDO gene sequence has a low homology (16%) with the IDO1, but their protein catalytic domains have a high similarity (60) and TDO is predominantly expressed in the liver (61). Similar to IDO1, upregulated IDO2 and TDO may be associated with immune escape in some types of tumors (53, 55, 62, 63). Previous studies have reported that TDO enhances the migration and invasion of glioblastoma and breast cancer cells *in vitro* and treatment with a TDO inhibitor significantly inhibits distant metastasis in mice (64–66). Furthermore, IDO2^-/-^ mice display a decreased tumor size compared with wild-type mice (67). Pharmacological inhibition of TDO reduces the number of lung tumor nodules in mice (68). Apparently, enhanced IDO2 and TDO expression and activity may promote the progression and metastasis of LC and their activity is indistinguishable (49). Similar to the function of IDO1, upregulated IDO2 expression is linked to worse prognosis in NSCLC (53). Therefore, IDO2 inhibitors may be valuable for targeting LC and IDO2 may be a biomarker for immunotherapy (69). Moreover, there is little information on whether IDO2 expression is associated with resistance to cisplatin in LC patients and what the value of IDO2 is in diagnosis and prognosis of LC (26, 63). Therefore, further studies are warranted to address these questions.

## Try Metabolites and Immune Escape In LC

**Figure 1:**
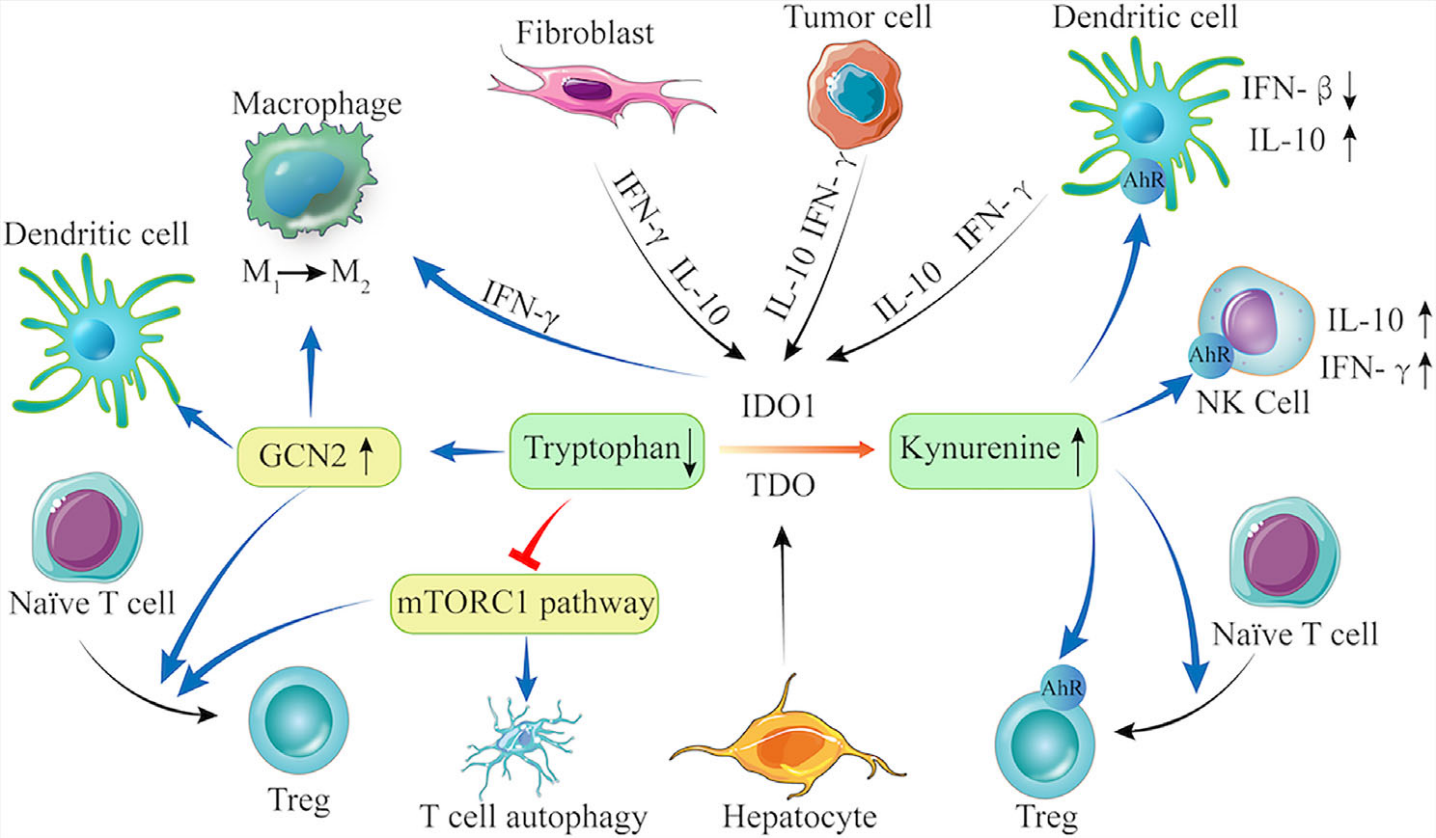
The Try-IDO1/TDO-KYN pathway and immune escape. IDO1 is constitutively expressed in fibroblast, tumor cells, and DCs, and can be upregulated by IL-10 and IFN-γ, whereas TDO is only expressed in hepatocytes. When IDO1 and TDO are activated, they promote Try degradation and KYN accumulation. Try depletion can activate GCN2 and inhibit the mTORC1 signaling. The KYN can bind to AhR in NK cells, Tregs, and DCs. Therefore, the Try-IDO1/TDO-KYN pathway cooperatively modulates immune cells (e.g., DCs, macrophage, Treg, and T cells) to regulate anti-inflammatory cytokine production, leading to enhanced immunosuppression in the tumor microenvironment.

The immune escape is a “hallmark of cancer” (70, 71). Tumor immune escape refers to the phenomenon, in which tumor cells can grow and metastasize by avoiding recognition and attack by the immune system through various mechanisms (72). Currently, IDO1 has been suggested to be important for immune escape of LC. First, upregulated IDO1 expression promotes the degradation of Try and the accumulation of its metabolites (such as KYN, 3-HAA and others) in LC. These metabolites act on various immune cells, including T cells (naive CD4+ T cells, Th17, and Treg), antigen-presenting cells (APC, DCs, and macrophages), and NK cells, and lead to immune escape. The promising mechanisms by which Try metabolites induce cancer immune tolerance and immunosuppression are summarized in **Figure 1**.

First, Try is an essential amino acid for immune cell proliferation, and Try depletion results in T-cell apoptosis, which is one major reason for cancer immunosuppression (73). The decreased Try levels can inhibit T-cell proliferation by activating general control over nonderepressible 2 (GCN2) kinase and suppressing the mTOR signaling, a target of rapamycin (74–76). The GCN2 is a serine/threonine kinase and can phosphorylate eukaryotic initiation factor 2a kinase (eIF2a) in the presence of low concentration of Try, inhibiting protein synthesis and T cell proliferation (74). Activated GCN2 can also promote the differentiation of naïve CD4+ T cells into Tregs (16, 74, 77). Furthermore, GCN2 can alter the phenotype of DCs and macrophages (75, 76, 78), making them prone to immunosuppression to promote tumorigenesis. In contrast, other studies argue that GCN2 is a sensor of amino acid starvation and its activation is not dependent on a low Try level, rather than deficiency in many amino acids (79, 80). Actually, T cells with GCN2 deficiency have similar activity to wild-type T cells in B16 melanoma-bearing mice (79), which contradicts the tumor promotion of GCN2. Apparently, there may be another mechanism that senses Try-deprived condition to regulate T cell immunity against tumor. The mTOR signaling appears to be a possible candidate (81, 82) because inhibition of mTOR complex 1 (mTORC1) can induce T-cell autophagy and anergy in the tumor microenvironment (83). Moreover, mTORC1 inhibition can also induce Treg cells to suppress anti-tumor immune responses (82).

Second, increased KYN can lead to immune tolerance by inhibiting T cell proliferation and inducing T cell apoptosis to promote tumor growth (38). The KYN is a ligand of AhR, and its activation promotes Treg cell differentiation that can directly inhibit anti-tumor immune responses, contributing to cancer immune escape (77, 84). The AhR activation can also direct DCs and macrophages toward an immune-suppressive phenotype (85–87). The AhR activation enhances IL-10 synthesis and secretion, and inhibits the IFN-β signaling in DCs, but induces IL-10 and IFN-α production in NK cells, respectively. Consistently, higher frequency of Tregs is detected in mice bearing cisplatin-resistant tumors than those bearing cisplatin-sensitive tumors (26).

Third, the downstream metabolites (such as 3-HAA and QA) of KYN can also induce T-cell apoptosis (88), contributing to immune tolerance. Recent studies have shown that QA can inhibit the proliferation of cancer-specific CD8+ cytotoxic T and NK cells to promote tumor growth (89). Furthermore, LC patients with lower plasma 3-HAA, the precursor of QA, benefit more from ICI treatment, suggesting that plasma 3-HAA levels may be a biomarker for predicting the response of LC patients to ICIs (23). The lower plasma 3-HAA may reflect less immunosuppression in patients because 3-HAA can promote Treg responses to produce high levels of TGF-β that decrease effector T-cell function, leading to immunosuppression (90). However, its precise mechanism in tumor immunity is not clear.

Next, IDO1 expression can be regulated by cytokines, such as IL-10 and IFN-γ (91, 92) while IDO1 inhibition can enhance T-cell proliferation and infiltration in the tumor environment and IL-2 production (93). Furthermore, IDO1 or IDO2 deficiency can modulate the tumor microenvironment by reducing KTR, enhancing immune cell infiltration and IFN-γ production (67). TDO and IDO2 act as the Try-metabolizing enzymes and can also promote Try degradation, resulting in immune regulation similar to IDO1. However, there are few reports and further studies are needed.

Last, IDO1 and TDO catalyze the production of several downstream Try metabolites, such as KYN (64, 84), KA (94), and XA (66), which can activate the AhR and may contribute to the immune modulation of IDO1 and TDO. Interestingly, KYN can directly bind and activate the AhR, with a high affinity at low picomolar levels (95). However, whether similar mechanisms also apply to other polar metabolites that activate the AhR, such as KA, remains to be investigated. In addition, AhR can regulate IDO-related regulatory phenotype in DCs (96). Here, an autocrine IDO−KYN/AhR−IDO feedback loop may contribute to the immune modulation (97, 98).

## The Clinical Applications of Try Metabolites in LC

Enhanced IDO1 expression and activity can evade immunosurveillance and are associated with poor prognosis of LC. Therefore, inhibition of IDO1 may be an ideal strategy for intervention of LC. There are several direct IDO1 inhibitors available, including epacadostat and navoximod that neither directly kill tumor cells, nor spontaneously initiate an immune response (99). Unlike epacadostat, the Try mimetic indoximod (D-1MT, 1-methyl-D-tryptophan) is the first non-enzyme inhibitory drug that targets the IDO1 pathway and can inhibit lung tumor growth *in vivo* (100–102). Indoximod can directly act on immune cells by creating an artificially Try-related signal, relieving the IDO1-mediated immunosuppression (99). There are ongoing clinical trials that investigate anti-IDO1 agents as monotherapy or adjuvant therapies with other drugs for various solid tumors. The clinical trials of anti-IDO1 agents for different combination strategies, such as combination with ICIs, other immunomodulators, and chemotherapy, are summarized in **Table 1**.

**Table 1:** Clinical trials for the potential drugs targeting the IDO1/TDO-KYN pathway.

Indication	Tumor type	Combination	Status	ClinicalTrials.gov	Phase
IDO inhibitor: Epacadostat	Metastatic NSCLC	Pembrolizumab	Complete	NCT03322540	II
	Metastatic NSCLC	Pembrolizumab and Platinum-based Chemotherapy	Completed	NCT03322566	II
	NSCLC	Nivolumab	Terminated	NCT03348904	III
	Extensive Stage Small Cell Lung Cancer	Pembrolizumab	Withdrawn	NCT03402880	II
	NSCLC, UC	Atezolizumab	Terminated	NCT02298153	I
	Advanced Solid Tumor, NSCLC	Sirolimus	Recruiting	NCT03217669	I
	Solid Tumor, Advanced Malignancies, Metastatic Cancer	Azacitidine and Pembrolizumab	Completed	NCT02959437	I/II
	Solid Tumor, Head and Neck Cancer, Lung Cancer, UC	Durvalumab (MEDI4736)	Completed	NCT02318277	I/II
	B-cell Malignancies, CRC, Head and Neck Cancer, LC, Lymphoma, Melanoma,Ovarian Cancer, Glioblastoma	Nivolumab	Completed	NCT02327078	I/II
	Microsatellite-instability High CRC, Endometrial Cancer, Head and Neck Cancer, HCC, GC, Lung Cancer, Lymphoma, RCC, Ovarian Cancer, Solid Tumor, UC, Melanoma, Bladder Cancer, TNBC	Pembrolizumab	Active, not recruiting	NCT02178722	I/II
	NSCLC	Pembrolizumab and chemotherapy	Completed	NCT02862457	I
	Solid Tumor	INCB001158 and Pembrolizumab	Terminated	NCT03361228	I/II
	Advanced Malignancies, Metastatic Cancer	INCAGN01876 and Immune Therapies	Completed	NCT03277352	I/II
	Solid Tumor	Pembrolizumab and Chemotherapy	Completed	NCT03085914	I/II
	Solid Tumor	Nivolumab and Immune Therapies	Active, not recruiting	NCT03347123	I/II
IDO inhibitor: Navoximod	Advanced solid tumor	–	Completed	NCT02048709	I
IDO inhibitor: BMS-986205	NSCLC	Nivolumab and Chemotherapy	Withdrawn	NCT03417037	III
	Advanced Cancer, Melanoma, NSCLC	Nivolumab and Ipilimumab	Recruiting	NCT02658890	I/II
IDO inhibitor: MK-7162	Advanced solid tumor	Pembrolizumab	Recruiting	NCT03364049	I
IDO inhibitor: LY3381916	LY3381916 alone or in combination with LY3300054 in solid tumors	LY3300054	Recruiting	NCT03343613	I
IDO inhibitor: KHK2455	Locally advanced or metastatic solid tumors	Mogamulizumab	Recruiting	NCT02867007	I
IDO pathway modulator: Indoximod (D-1-MT)	NSCLC, Progression of NSCLC, NSCLC Recurrent	Tergenpumatucel-L and docetaxel	Active, not recruiting	NCT02460367	I
	Metastatic or refractory solid tumors	N/A	Completed	NCT00567931	I
	Relapsed or Refractory Solid Tumors	–	Terminated	NCT00739609	I
IDO pathway modulator: NLG-802	Advanced solid tumors	N/A	Recruiting	NCT03164603	I
Dual IDO1/TDO inhibitor: HTI1090/SHR9146	Advanced solid tumors	SHR-1210 and apatinib	Not yet recruiting	NCT03491631	I
	Advanced solid tumors	N/A	Recruiting	NCT03208959	I
IDO Peptide Vaccination	NSCLC	–	Completed	NCT01219348	I

Data accessed from https://www.clinicaltrials.gov/ on January 15, 2021. UC, urothelial cancer; CRC, colorectal cancer; HCC, hepatocellular carcinoma; RCC, renal carcinoma; N/A, not applicable.

Epacadostat, a small-molecule IDO1 inhibitor, was developed by Incyte and is being tested for its therapeutic efficacy and safety in an advanced stage of clinical trial (103). The phase I/II KeyNote 037/ECHO 301 trial to test the safety and efficacy of different doses of epacadostat combined with 200 mg pembrolizumab (i.e., an anti-PD1 agent) every 3 weeks (Q3W) in 62 patients with different types of cancers has achieved promising results (104). There were 24% of patients experiencing high-grade toxicities but no treatment-related death and 12 out of 22 patients obtained objective responses. Unfortunately, a further phase III clinical trial with epacadostat 100 mg twice a day (BID) and pembrolizumab 200 mg (Q3W) failed to improve progression-free survival (PFS) in patients with metastatic melanoma (105). Because of the limitations of this trial, further clinical trials are necessary to test its therapeutic efficacy and safety.

The phase I ECHO-110 study was designed to test epacadostat at different doses combined with atezolizumab (i.e., an anti-PD-L1 agent) 1,200 mg Q3W in 29 patients with stage IIIB/IV NSCLC, who had previously been treated with ≥1 prior line of platinum-based chemotherapy (≥2 cycles), but not with checkpoint/IDO inhibitors. Similarly, 7 out of 29 patients displayed high-grade toxicities but no treatment-related death. Epacadostat at a dose up to 300 mg BID combined with atezolizumab 1,200 mg Q3W was well-tolerated in patients with previously treated NSCLC (103). However, only one patient achieved objective response. The low therapeutic efficacy may stem from the fact of almost all patients with negative PD-L1 expression. Similarly, the single-arm combination of the ECHO-301 trial also failed, lining with the results from other Phase II and III trials conducted in different settings (17) and was converted into the randomized phase II trials of epacadostat combined with pembrolizumab in LC patients. In addition, the KEYNOTE-715-06/ECHO-306-06 trials with the combination of epacadostat, pembrolizumab, and platinum-based chemotherapy did not obtain promising benefit in overall response rate in NSCLC patients (Clinicaltrial.gov.). These observations suggest that the combination of Epacadostat and a PD-1/PD-L1 blockade may not be valuable for patients with PD-L1 negative LC. However, whether this treatment strategy can achieve positive responses in PD-L1 expressing NSCLC or whether combination with platinum-based chemotherapy can achieve a better outcome in NSCLC patients has not been clarified. The ongoing, randomized, phase 2 ECHO-305 (NCT03322540) and ECHO-306 (NCT03322566) trials may give promising results.

New IDO inhibitors, such as navoximod (NLG-919/GDC-0919) and BMS-986205, are also being tested in clinical trials (106). In a phase I study of the IDO1 inhibitor, combination of navoximod and atezolizumab displayed acceptable safety, tolerability, and pharmacokinetics, but not clear beneficial evidence of navoximod in patients with advanced solid tumors (107).

There are questions on whether epacadostat doses used in the ECHO-301 trial could effectively inhibit IDO1 activity in the tumor microenvironment and whether targeting multiple enzymes in the KYN pathway to control Try metabolism would benefit to these patients (58).

There are also ongoing trials testing IDO1 and TDO dual inhibitors such as HTI-1090 (SHR9146) as a monotherapy for solid tumors (NCT03208959). The dual inhibitor of DN1406131 is being tested for its safety in healthy subjects (NCT03641794) while RG70099 from Curadev/Roche is still in preclinical development (108).

In a word, most researchers have focused on IDO/TDO inhibitors for the treatment of LC, and some of them have already been tested in clinical trials. However, the current therapeutic efficacy appears limited. Thus, further studies are necessary to understand the biological functions of Try and its metabolites in the development and progression of LC. Given that the KYN downstream metabolites have profound functions in regulating T cell immunity against LC, these metabolites and their catalyzing enzymes may be explored for development of therapies for LC. Similarly, combination of IDO/TDO inhibitors and other therapies (chemotherapy, radiotherapy, targeted therapy, and immunotherapy) should be pursued to determine the safety and therapeutic efficacy in LC. Previous studies have demonstrated that patient’s metabolism (BMI variation and hypercholesterolemia) has a significant impact on the outcome of PD1 inhibitor treatment in LC patients (109, 110). Some drugs can regulate body metabolism and are significantly related to clinical outcomes of ICI treatment in LC patients (111, 112). Metformin, an effective agent for the management of type 2 diabetes mellitus, in combination with ICI treatment can improve the anticancer effects of ICIs (113, 114). Statins can inhibit cholesterol production (115) and is associated with better clinical outcome of anti-PD1 treatment in advanced NSCLC patients in an intensity-dependent manner (111). However, IDO1 as an immune checkpoint is not as well studied as PD1, and the role of patient metabolism and drugs involved in its regulation on the outcome of patients treated with IDO/TDO inhibitors needs to be further confirmed. If demonstrated, IDO/TDO inhibitors may benefit many patients with LC.

## Conclusion

Currently, modulation of Try metabolism has been used for diagnosis, prognosis, and therapies for LC. The levels of circulating IDO activity and downstream metabolites (3-HAA, QA, KA, etc.) can be used to predict the efficacy of different treatments in LC (116, 117). However, the results are inconsistent, which may be caused by limitations, such as small sample size, inconsistent measurement methods, influence of the gender, tumor stage, and tumor heterogeneity. Hence, further studies are needed in multi-centers with a larger population, standardized measurement methods, paired samples, and detailed analysis for different stages and pathological types of LC. Currently, some metabolites, enzyme inhibitors targeting immune checkpoints, and modulators have been developed for the diagnosis and treatment of LC. Because the change in metabolomics is one of the factors for the development of cancer, it will be wise to integrate the role of metabolomic changes in the pathogenesis of LC and consider other factors together for the development of therapeutic strategies for LC. Therefore, further studies are necessary to understand the process of complicated Try metabolism and its regulation in different types and stages of LC.

## Author Contributions

CL performed the literature search and drafted the manuscript and figur**e**s. HZ edited and revised the manuscript. All authors contributed to the article and approved the submitted version.

## Funding

This work was supported by grants from the National Natural Science Foundation of China 81703087, the United Fund of the Second Hospital of Dalian Medical University and Dalian Institute of Chemical Physics, Chinese Academy of Sciences (UF-ZD-202011), and the Project of Education Department of Liaoning Province (LZ2020009).

## Conflict of Interest

The authors declare that the research was conducted in the absence of any commercial or financial relationships that could be construed as a potential conflict of interest.

## Publisher’s Note

All claims expressed in this article are solely those of the authors and do not necessarily represent those of their affiliated organizations, or those of the publisher, the editors and the reviewers. Any product that may be evaluated in this article, or claim that may be made by its manufacturer, is not guaranteed or endorsed by the publisher.
